# The effect of culinary interventions (cooking classes) on dietary intake and behavioral change: a systematic review and evidence map

**DOI:** 10.1186/s40795-019-0293-8

**Published:** 2019-05-10

**Authors:** Bashar Hasan, Warren G. Thompson, Jehad Almasri, Zhen Wang, Sumaya Lakis, Larry J. Prokop, Donald D. Hensrud, Kristen S. Frie, Mary J. Wirtz, Angela L. Murad, Jason S. Ewoldt, M. Hassan Murad

**Affiliations:** 1 Evidence-Based Practice Research Program, Mayo Clinic, Rochester, MN USA; 2 Robert D. and Patricia E. Kern Center for the Science of Health Care Delivery, Mayo Clinic, Rochester, MN USA; 3 Division of Preventive, Occupational, and Aerospace Medicine, Mayo Clinic, 200 1st St SW, Rochester, MN 55905 USA; 4 Mayo Clinic Libraries, Mayo Clinic, Rochester, MN USA; 5 Dan Abraham Healthy Living Center, Mayo Clinic, Rochester, MN USA

**Keywords:** Cooking classes, Culinary intervention, Dietary intake, Nutrition, Systematic review, Evidence map, Chronic disease prevention

## Abstract

**Background:**

Culinary interventions (cooking classes) have been used to improve the quality of dietary intake and change behavior. The aim of this systematic review is to investigate the effects of culinary interventions on dietary intake and behavioral and cardiometabolic outcomes.

**Methods:**

We conducted a systematic review of MEDLINE, EMBASE, Cochrane Central Register of Controlled Trials, Cochrane Database of Systematic Reviews, and Scopus for comparative studies that evaluated culinary interventions to a control group or baseline values. The intervention was defined as a cooking class regardless of its length or delivery approach. Studies included populations of children, healthy adults or adults with morbidities. The risk of bias was assessed using the Cochrane Risk of Bias tool and the Newcastle-Ottawa Scale. Outcomes were pooled using the random-effects model and descriptive statistics and depicted in an evidence map. Simple logistic regression was used to evaluate factors associated with intervention success.

**Results:**

We included 30 studies (6 were randomized, 7381 patients, average follow up 25 weeks). Culinary interventions were not associated with a significant change in body mass index (− 0.07 kg/m^2^, 95% CI: -1.53, 1.40), systolic (− 5.31 mmHg, 95% CI: -34.2, 23.58) or diastolic blood pressure (− 3.1 mmHg, 95% CI: -23.82, 17.62) or LDL cholesterol (− 8.09 mg/dL, 95% CI: -84.43, 68.25). Culinary interventions were associated with improved attitudes, self-efficacy and healthy dietary intake in adults and children. We were unable to demonstrate whether the effect of a culinary intervention was modified by various characteristics of the intervention such as its delivery or intensity. Interventions with additional components such as education on nutrition, physical activity or gardening were particularly effective.

**Conclusions:**

Culinary interventions were not associated with a significant change in cardiometabolic risk factors, but were associated with improved attitudes, self-efficacy and a healthier dietary intake in adults and children.

**Electronic supplementary material:**

The online version of this article (10.1186/s40795-019-0293-8) contains supplementary material, which is available to authorized users.

## Background

Multiple prospective studies have demonstrated the benefits of consuming more fruits/vegetables, whole grains, and nuts and less red meat and sweets/desserts [1]. Yet, studies also show that most people continue to eat a suboptimal diet. Fewer than 20% of American children and adults are eating enough fruits and vegetables [2]. The American Heart Association reports diet as poor in 70 to 80% and ideal in less than 1% of Americans [3]. Thus, there is an urgent need for programs that result in changes in eating habits.

One potentially innovative approach is to provide individuals in need for behavioral change with demonstration or participation cooking classes. Usually such classes are taught by or with a dietitian and can involve nutrition education as well. A class can be a cooking demonstration, but many classes involve hands on cooking along with eating the food prepared. Such classes often provide needed skills such as how to prepare vegetables in a quick and appetizing manner. By having participatory cooking and eating, it is hoped that children and adults will increase their intake of healthy food and decrease their intake of unhealthy food. However, it is unclear if such classes result in significant changes in eating behavior. Therefore, we performed a systematic review and meta-analysis of the literature on cooking classes and eating behaviors to investigate the effects of culinary interventions on dietary intake, behavioral change, cardiometabolic outcomes, anthropometric measures and quality of life. We included healthy and morbid participants from all ages. We included cooking classes of any duration or delivery approach. The studies included were comparative to a control group or to baseline values. We also developed an evidence map, which is a visual depiction of the state of the evidence that can be used by policymakers for decision making or setting a future research agenda [4].

## Methods

The reporting of this systematic review complies with the Preferred Reporting Items for Systematic Reviews and Meta-Analyses (PRISMA) statement [5] and follows a priori established protocol.

### Data sources and search strategies

A comprehensive search of several databases from 1990 to May 5th, 2017, English language was conducted. The databases included MEDLINE, Epub, Ahead of Print, Medline In-Process & Other Non-Indexed Citations, EMBASE, Cochrane Central Register of Controlled Trials, Cochrane Database of Systematic Reviews, and Scopus. The search strategy was designed and conducted by a medical reference librarian with input from the study investigators. Controlled vocabulary supplemented with keywords was used to search for studies of interventions utilizing cooking classes. The actual strategy is available in Additional file 1: Appendix.

### Study selection

We included studies in which the intervention was a cooking class (i.e., culinary intervention) and outcomes were compared after a follow-up period to a control group or baseline (i.e., pre-post). Study designs included were randomized clinical trials (RCTs) and nonrandomized trials (including cohort and pre-post studies). The intervention was defined as a cooking class regardless of the number of sessions, or whether it was delivered by a chef, an educator or a dietitian. Studies included populations of children, healthy adults, or adults with morbidities. Multicomponent studies were included as long as components added to cooking classes were two or less. Studies which had more than 2 additional intervention components were excluded because of the limitation of attributing effects to multiple components.

Outcomes of interest were anthropometrics measures, cardiometabolic outcomes, behavioral outcomes, dietary intake and quality of life.

**Fig. 1:**
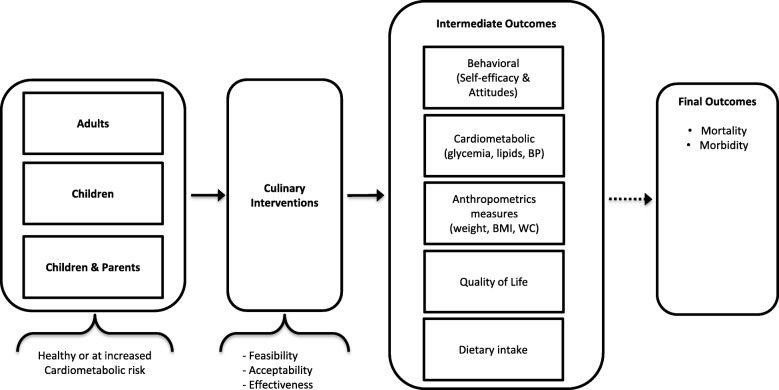
Analytic framework for the plausible effects of culinary interventions

Figure 1 depicts an analytic framework of the current study showing the effects of culinary interventions on the studied intermediate outcomes and possible effect on clinically important patient outcomes.

We did not restrict time or study location. We excluded editorials, letters, systematic reviews, and errata. Independent reviewers, working in duplicates, screened the titles and abstracts of all citations and then the full text of eligible references. Discrepancies between the reviewers were resolved through discussions and consensus. If consensus was not reached, a third reviewer was asked to resolve the difference.

### Data extraction and risk of Bias assessment

We developed pilot-tested standardized data extraction forms. The following information was extracted: author, study design, population, study purpose, inclusion and exclusion criteria, intervention duration, intervention components and description, number of sessions, cooking class provider, control description, whether the study was participatory or not, sample size, follow-up duration and outcomes. We used the Cochrane Risk of Bias tool to assess the risk of bias in RCTs [6]. The overall risk of bias across the various domains was determined by focusing on random sequence generation, allocation concealment, blinding of participants or personnel, blinding of outcome assessment, incomplete outcome data, and reporting bias or selective reporting. For observational studies, we selected items from the Newcastle-Ottawa Scale, with focus on the representativeness of the exposed cohort, selection of the non-exposed cohort, ascertainment of exposure and outcomes, comparability of cohorts and adequacy of follow-up [7]. Data extraction and quality assessment were completed by pairs of independent reviewers.

### Outcome measures

We evaluated two types of outcomes, cardiometabolic outcomes and behavioral outcomes. The cardiometabolic outcomes included glucose, hemoglobin A1c (HbA1c), insulin, homeostatic model assessment for insulin resistance (HOMA-IR), total cholesterol, triglycerides, low density lipoprotein cholesterol (LDL-C), high density lipoprotein cholesterol (HDL-C), systolic blood pressure (SBP), diastolic blood pressure (DBP) and anthropometrics measures (body mass index (BMI), waist circumference and body fat percentage). Behavioral outcomes included attitudes, self-efficacy, and healthy dietary intake.

Dietary intake, being a complicated multifaceted outcome, was assessed as healthy intake, which was defined as any healthy change in dietary intake (increase in favorable food groups (fruits, vegetables, low-fat dairy and whole grains) and other sources of dietary fiber, lean sources of protein and unsaturated fats or decrease in unfavorable ones (fast food, high carbohydrate foods, high sugar desserts, saturated and trans fats, and high sodium foods)).

### Data synthesis and analysis

For outcomes that were reported with adequate data to allow for meta-analysis (i.e., mean and dispersion measures), we used the DerSimonian and Laird random-effects model [8] to pool the mean differences across studies (BMI, SBP, DBP, LDL). I^2^ was used to evaluate heterogeneity, in which > 50% suggests substantial heterogeneity [9, 10]. To evaluate factors affecting the overall success of the intervention, we dichotomized self-efficacy healthy intake and -specifically- fruit and vegetable intake outcomes: 1 shows significant improvement; and 0 shows nonsignificant or negative improvement. Emphasis was placed on fruit and vegetable intake because it was the dietary intake measure most frequently studied. Simple logistic regression models were then used to evaluate associated factors, including sample size, cooking class provider, population (children or adults), whether the cooking class was participatory, number of sessions, and the intervention duration. All statistical analyses were conducted using Stata version 15.1 (StataCorp LLC, Station College, TX).

In cases were meta-analysis was not feasible because too few studies reported the outcome with sufficient details to allow statistical analysis, we presented the results narratively.

We presented all study outcomes (quantitative and qualitative) in an evidence map [11]. An evidence map shows the overall effects of culinary interventions along with the risk of bias and certainty in these effects, helping decision makers understand the possible benefits and gaps in research. The certainty in evidence was rated using the GRADE approach [12] and narrative adaptations [13].

## Results

### Study characteristics

**Fig. 2:**
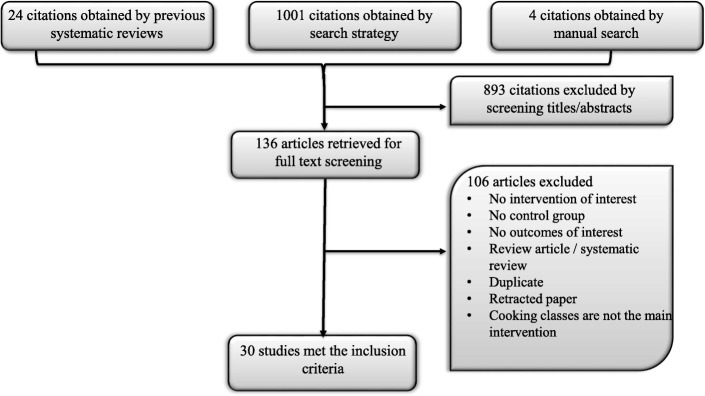
Flow chart depicting the process of study selection

The Search strategy (done in May, 2017) identified 1001 relevant citations. 28 additional studies were identified through asking clinicians with expertise about the topic area and reference mining. A total of 30 unique studies met the inclusion criteria enrolling 7381 patients (Fig. 2). Adults were enrolled in 14 studies [14–27], children in 12 studies [28–39], and both age groups in 4 studies [40–43]. The majority (17) were nonrandomized controlled studies [15, 17–21, 26, 27, 29–33, 35, 36, 38, 39]. There were 7 pre-post studies [14, 16, 23, 28, 37, 41, 42] and 6 RCTs [22, 24, 25, 34, 40, 43].

### Description of the intervention

Cooking classes ranged in duration from 2 weeks to 104 weeks (2 years), with a mean duration of 21 weeks, whereas the number of sessions ranged from 1 session to 52 sessions, with a mean of 8 sessions. The class instructor was a chef in 9 studies [17, 18, 20, 22, 26, 29, 33, 35, 39], a dietitian in 10 studies [14, 15, 19, 21, 23–25, 28, 40, 42], an educator in 7 studies [27, 30–32, 34, 36, 38], and 4 studies did not report the class instructor. In 21 studies, the cooking classes were participatory [14, 15, 19–22, 24, 26, 28, 32–43], whereas 3 studies featured a cooking demonstration [16, 18, 30] and 6 studies were not clear in that aspect. Additional file 1: Table S1 lists the included studies’ characteristics.

The mean follow-up duration was 25 weeks. 27 studies reported a behavioral outcome [15–20, 22–24, 26–43], whereas 11 studies reported cardiometabolic outcomes and anthropometrics measures [16, 17, 19, 21, 22, 24, 25, 34, 38, 42, 43], and 2 studies reported on quality of life [15, 21]. Additional file 1: Table S2 lists the outcomes evaluated in each included study.

### Risk of Bias

Six RCTs were assessed using the Cochrane Risk of Bias tool. All 6 RCTs have a high risk of bias, mainly because of inadequate procedures for randomization, concealment and blinding, or poor reporting of how these design features were achieved. The assessment of these studies is listed in Additional file 1: Table S3.

The remaining 24 studies were assessed using items from the Newcastle-Ottawa Scale. 17 studies were Nonrandomized controlled trials and 7 studies were Pre-Post studies. 6 studies had a high risk of bias, and 19 studies had a moderate risk of bias. Most studies were deficient in the component of comparability of the design or analysis (matching or confounder adjustment) and in the assessment of outcomes which relied on self-report in most cases and were subject to recall bias or interviewer bias (reporting bias). Additional file 1: Table S4 lists the risk of bias assessment for these 24 studies.

### Effect on anthropometrics and cardiometabolic outcomes

Meta-analysis did not show a significant effect of culinary interventions on BMI, SBP, DBP or LDL-C.

**Fig. 3:**
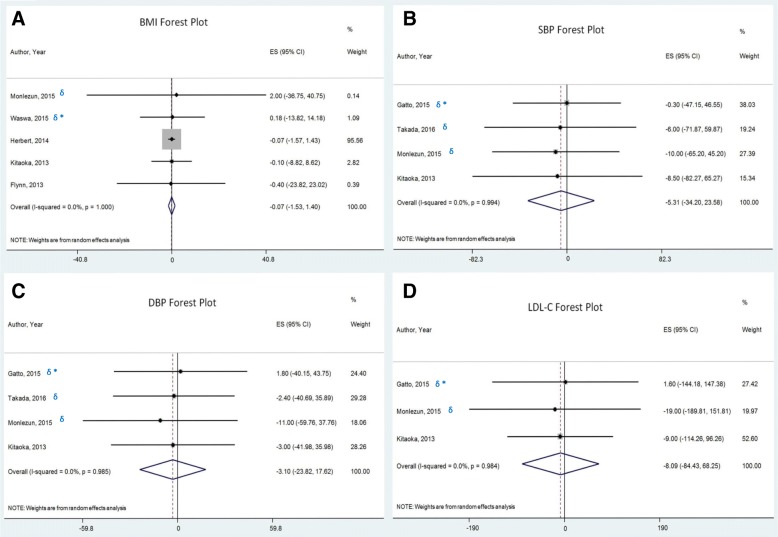
Forest Plots representing the overall mean difference and associated 95% confidence interval (CIs; horizontal lines) of (**a**). Body mass index (BMI), (**b**). Systolic blood pressure (SBP), (**c**). Diastolic blood pressure (DBP), (**d**). Low-density lipoprotein cholesterol (LDL-C). The gray squares the weights used in the meta-analysis. The asterisk (*) represents studies in children and the sign (δ) represents randomized clinical trials (RCTs)

Figure 3.a depicts the meta-analysis of BMI. Five studies [16, 17, 19, 22, 43] were analyzed for this outcome, with an overall population size of 1292 persons. The mean follow up duration was 30 weeks. The overall mean difference was − 0.07 kg/m^2^ (95% CI: -1.53, 1.40).

Figures 3.b and 3.c illustrate the meta-analysis of SBP and DBP, respectively. Four studies [19, 22, 25, 34] were analyzed for these outcomes, with an overall population size of 478 persons. The mean follow up duration was 15 weeks. The overall mean difference was − 5.31 mmHg (95% CI: -34.2, 23.58) for SBP and − 3.1 mmHg (95% CI: -23.82, 17.62) for DBP.

Figure 3.d represents the meta-analysis of LDL-C. Three studies [19, 22, 34] were analyzed for this outcome, with an overall population size of 410 persons. The mean follow up duration was 17 weeks. The overall mean difference was − 8.09 mg/dL (95% CI: -84.43, 68.25).

Only one study [34] evaluated the outcomes of glucose, insulin, and insulin resistance (HOMA-IR). None of these outcomes significantly changed (*p* values of 0.56, 0.88 and 0.85, respectively). Only one study evaluated the outcomes of HbA1c [22] which also did not change significantly (*p* = 0.58).

### Attitudes, self-efficacy and dietary intake

Data on attitudes, self-efficacy and dietary intake were not reported in a way to allow quantitative analysis; therefore, these outcomes were reported narratively.

Participants’ attitudes (e.g. *how likely are you to eat the following foods?* [33] or *eating healthy is important to me* [20]) improved in adults and children (medium risk of bias).

Self-efficacy (e.g., *do you believe you can eat correct portions?* [22]) also improved in adults and children (medium risk of bias).

Both children and adults had improved healthy dietary intake after culinary interventions (medium risk of bias). For instance, Newman, 2005 [23] found a significant increase in total daily vegetable, fruit and fiber intake, as well as a significant decrease in fat intake after a cooking classes intervention. Quality of life in adults may have improved after the intervention in adults (2 small nonrandomized studies with medium risk of bias).

We conducted regression analysis to explore the effect of several possible effect modifiers on the success of the intervention. This analysis demonstrated no significant associations between the success of the intervention and sample size, cooking class provider, population (children vs. adults), whether the class was participatory (vs. demonstration), number of sessions, and the intervention duration. Results are depicted in Additional file 1: Table S5.

### Interventions with additional components

Several studies employed multicomponent interventions. The additional components were gardening education, dietary education, physical activity recommendations, goal setting and grocery store tours. These studies are summarized in Additional file 1: Table S6. In general, these studies showed statistically significant improvements in participants’ self-efficacy, dietary intake and attitudes. In one study, Curtis et al. [40] performed a randomized cluster trial in which 169 families (with 589 individuals) were randomized to 3 groups: nutrition education, cooking classes, and cooking classes + nutrition education + goal setting. The authors analyzed fat, carbohydrate, protein, vitamin C, and iron intake as well as energy density. There was a substantial drop out rate (25% at 3 months and 60% at 18 months). Fat intake decreased and carbohydrate intake increased (this was the goal of the project) more in the combination group than in the education group. Cooking classes alone had an intermediate effect (not statistically different from either group). Differences were not sustained at 18 months. Energy density (calculated and expressed as KJ/g of food) was significantly lower in the combination group at 18 months, but not at 3 months.

Gatto et al. [34] performed a randomized cluster trial in which 4 elementary schools (319 students) were randomized to a control group or an intervention which consisted of 12 ninety minute sessions. 45 min were gardening/nutrition lessons and 45 min were cooking/nutrition lessons. There were significantly greater declines in BMI and waist circumference in the experimental group versus the control group. There was also a difference in fiber intake between the two groups (with a small increase in the experimental group and a large decline in the control group). Correction for multiple comparisons was not done because this was a preliminary study.

### Evidence map

**Fig. 4:**
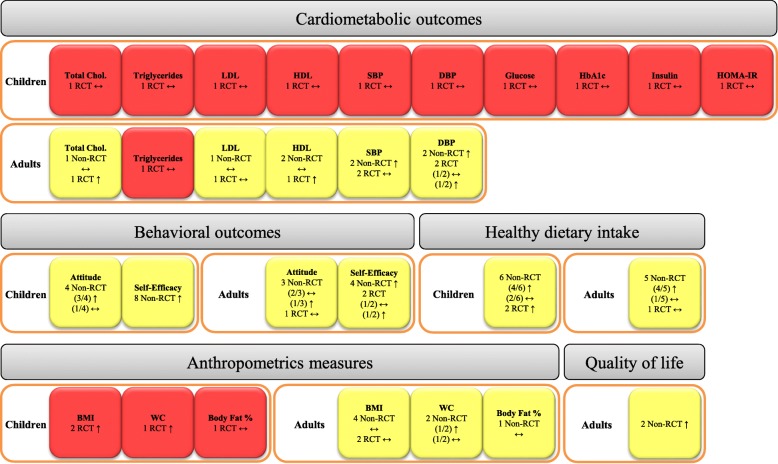
Evidence map showing the effects of culinary interventions. ↑: significant improvement, ↔: No significant change, ↓: significant worsening. Colors reflect risk of bias (red is high and yellow is medium and green is low), BMI: Body mass index, DBP: Diastolic blood pressure, HbA1c: Hemoglobin A1c, HDL: High-density cholesterol, HOMA-IR: Homeostatic Model Assessment for Insulin Resistance, LDL: Low-density cholesterol, RCT: Randomized controlled trial, SBP: Systolic blood pressure, WC: Waist circumference

To summarize quantitative and qualitative results, the various outcomes of this systematic review are presented in an evidence map demonstrating the effect of culinary interventions, the risk of bias, and study design contributing to each outcome (Fig. 4).

## Discussion

### Summary of evidence

We conducted a systematic review and meta-analysis and developed an evidence map to summarize the effect of culinary interventions on various outcomes. In brief, culinary interventions were not associated with statistically significant changes in BMI, SBP, DBP or LDL-C, but were associated with improved attitudes, self-efficacy and healthy dietary intake in adults and children. We were unable to demonstrate whether the effect of a culinary intervention is modified by various characteristics of the intervention such as its delivery or intensity. Studies with multiple components, particularly those deemed to have the lowest risk of bias, suggested significant improvement in attitudes, self-efficacy and dietary intake when the intervention had an additional component such as education on nutrition, gardening or physical activity.

### Limitations and strengths

The available literature suffers from important biases such as selection bias and high attrition rate. Synthesis of the evidence is also limited by clinical heterogeneity of the available interventions, population studied and outcomes. The evidence map suggests the need for evaluation of the intervention on intermediate markers of cardiovascular risk such as BMI, BP and lipids.

The strengths of this systematic review include the multidisciplinary nature of the research team, the comprehensive literature search, the duplicate process of selecting and appraising studies and the attempt to evaluate outcomes qualitatively and quantitatively.

### Practical implications

The current evidence suggests a benefit of culinary interventions. Despite the limitations in the literature and the lack of data to support an effect on cardiometabolic outcomes, which would require larger trials with longer follow up duration, it seems that improvements in behavior and attitudes are quite likely. In addition, if one considers the magnitude of change in some parameters such as blood pressure (5 mmHg observed in some studies), this change is clinically important despite the fact that it was not statistically significant. For practical purposes, one can view these findings by asking these two questions: Who are the individuals that are likely to benefit from such interventions? And; what logistical issues and barriers should be considered when implementing such programs?

### Candidates for the intervention

The ideal candidates for a culinary intervention are individuals who have a high motivation and desire to cook at home, but lack the necessary skills or have limited self-efficacy. Some of these skills that can be taught in a cooking class may bridge this gap and can include: knife handling, meal-planning, grocery shopping, food budgeting, prepping and cross utilizing ingredients, cooking techniques (searing, roasting, etc.), label reading and proper food storage. While these classes can be delivered in a one-on-one fashion, they are more commonly given in a group setting. Group-based cooking classes are not a good fit for everyone and require a level of homogeneity of audience in terms of kitchen skillsets, age and common interests (e.g., interest in healthy eating, a certain type of cuisine, quick meals, using specific kitchen equipment, budget-friendly foods, or more advanced culinary techniques).

### Barriers and logistics

Barriers for successful implementation of programs offering culinary interventions include the lack of consistent health insurance coverage, the need for appropriate marketing and advertisement, forecasting ingredient and staffing needs, and tailoring the class content to fit participant’s needs and desires while ensuring engagement of all participants. Prior to implementation, class instructors should conduct a needs assessment that determine the budget (labor cost, equipment cost, and ingredient cost), time constraints (for the cooking class itself and also for planning), equipment availability, space and venue, participant demographics, participant recruitment, food safety and sanitation, first aid and safety, group size, class fee structure and affordability, and group dynamics.

From a nutrition perspective, providing nutrition education in combination with the cooking class (before, during, and after the cooking class) may provide individuals with a more expansive knowledge base of how to replicate meals at home, while focusing on healthy nutrition patterns that incorporate more nutrient-dense foods, such as fruits and vegetables. A non-exhaustive list of nutrition education topics to provide may include: energy-dense foods versus nutrient-dense foods, the benefits of increasing nutrient-dense foods, healthy pantry staples, budget-friendly grocery shopping, meal preparation and cross utilizing ingredients, basic kitchen equipment, cooking conversions, and healthy recipe substitutions.

Cooking classes can be taught by a variety of individuals; Chefs, Registered Dietitians, a health or nutrition educator, or volunteers. At the present time, there is not clear evidence to determine who are the most effective instructors or which class type is the best (demonstration style intervention vs hands-on cooking intervention), or to determine a certain duration or number of classes. Therefore, these determinations should be made based on feasibility and audience needs.

Aside from the interventional literature summarized in this systematic review, many other studies link eating at home to healthier nutrition and lower food costs. An association between consuming home prepared meals and adhering to healthier diets has been demonstrated. Although this association was of a cross-sectional nature, individuals who ate home prepared meal were more likely to adhere to the DASH and the Mediterranean diet, consume fruits and vegetables, and have higher vitamin C plasma levels [44]. The Seattle Obesity Study showed that frequent at-home cooking was associated with higher Healthy Eating Index and reduced per capita food expenditures [45]. Therefore, a rationale for culinary interventions exists although the social determinants of home cooking are complex and include multiple social and cultural factors [46] aside from what a cooking class can offer.

## Conclusions

Culinary interventions were not associated with a significant change in cardiometabolic risk factors, but were associated with improved attitudes, self-efficacy, and a healthier dietary intake in adults and children. Interventions with additional components such as education on nutrition, gardening, or physical activity may be more effective.

## Additional file

Additional file 1: Appendix**.** Search Strategy**. Table S1**. Studies Characteristics. **Table S2**. Outcomes reported in individual studies. **Table S3**. Risk of bias assessment, randomized trials. **Table S4**. Risk of bias assessment, nonrandomized studies. **Table S5**. Possible effect modifiers of the effect of interventions. **Table S6**. Studies with intervention components in addition to cooking classes. (DOCX 1953 kb)

## Abbreviations

BMI: body mass index
DBP: diastolic blood pressure
HbA1c: hemoglobin A1c
HDL-C: high density lipoprotein cholesterol
HOMA-IR: homeostatic model assessment for insulin resistance
LDL-C: low density lipoprotein cholesterol
RCTs: randomized clinical trials
SBP: systolic blood pressure
