# Supplementary material for: The effect of culinary interventions (cooking classes) on dietary intake and behavioral change: a systematic review and evidence map
Source: BMC Nutr. 2019 May 10;5:29. doi: 10.1186/s40795-019-0293-8 (PMC7050805; doi:10.1186/s40795-019-0293-8)
Supplement: Supplementary file 1 — Appendix. Search Strategy. Table S1. Studies Characteristics. Table S2. Outcomes reported in individual studies. Table S3. Risk of bias assessment, randomized trials. Table S4. Risk of bias assessment, nonrandomized studies. Table S5. Possible effect modifiers of the effect of interventions. Table S6. Studies with intervention components in addition to cooking classes. (DOCX 1953 kb) [file 40795_2019_293_MOESM1_ESM.docx]

| **The Effect of Culinary Interventions (Cooking Classes) on Dietary Intake and Behavioral Change: A Systematic Review and Evidence Map. (Appendix)** |
| --- |
|  |
| Bashar Hasan, MD * Warren G. Thompson, MD* Jehad Almasri, MD Zhen Wang, PhD Sumaya Lakis, MD Larry J Prokop, MLS Donald D. Hensrud , MD, MPH Kristen S. Frie, RDN, LD Mary J. Wirtz, RDN, LD Angela L. Murad, RDN, LD Jason S. Ewoldt, RDN, LD M. Hassan Murad MD, MPH  ^*^ First and second author contributed equally to this report. |

Table of Contents

[*Search Strategy* 3](#_Toc518909982)

[*Supplemental Figures* 8](#_Toc518909983)

[*Supplemental Tables* 12](#_Toc518909984)

[*Table 1: Studies Characteristics* 12](#_Toc518909985)

[*Table 2: Outcomes reported in individual studies* 19](#_Toc518909986)

[*Table 3: Risk of bias assessment, randomized trials* 24](#_Toc518909987)

[*Table 4: Risk of bias assessment, nonrandomized studies* 25](#_Toc518909988)

[*References* 27](#_Toc518909989)

Appendix

# *Search Strategy*

Ovid

Database(s): Embase 1988 to 2017 Week 19, EBM Reviews - Cochrane Central Register of Controlled Trials April 2017, EBM Reviews - Cochrane Database of Systematic Reviews 2005 to May 4, 2017, Ovid MEDLINE(R) Epub Ahead of Print, In-Process & Other Non-Indexed Citations, Ovid MEDLINE(R) Daily and Ovid MEDLINE(R) 1946 to Present
Search Strategy:

| **#** | **Searches** | **Results** |
| --- | --- | --- |
| 1 | exp Cooking/ | 22692 |
| 2 | exp Cooking/ed [Education] | 1 |
| 3 | exp Education/ | 1746084 |
| 4 | 1 and 3 | 1624 |
| 5 | (((cooking or "food preparation" or culinary) adj3 (class* or instruction or education or intervention* or program* or course* or workshop*)) or "teaching kitchen").ti,ab,hw,kw. | 830 |
| 6 | 2 or 4 or 5 | 2205 |
| 7 | exp evidence based medicine/ | 999874 |
| 8 | exp meta analysis/ | 203909 |
| 9 | exp Meta-Analysis as Topic/ | 49191 |
| 10 | exp "systematic review"/ | 133370 |
| 11 | exp Guideline/ or exp Practice Guideline/ | 429832 |
| 12 | exp controlled study/ | 5552484 |
| 13 | exp Randomized Controlled Trial/ | 889447 |
| 14 | exp triple blind procedure/ | 157 |
| 15 | exp Double-Blind Method/ | 394365 |
| 16 | exp Single-Blind Method/ | 67049 |
| 17 | exp latin square design/ | 334 |
| 18 | exp Placebos/ | 316532 |
| 19 | exp Placebo Effect/ | 9625 |
| 20 | exp comparative study/ | 2727096 |
| 21 | exp intervention studies/ | 31746 |
| 22 | exp Cross-Sectional Studies/ | 454886 |
| 23 | exp Cross-Over Studies/ | 123679 |
| 24 | exp Cohort Studies/ | 2093479 |
| 25 | exp longitudinal study/ | 324879 |
| 26 | exp retrospective study/ | 1167810 |
| 27 | exp prospective study/ | 898732 |
| 28 | exp population research/ | 81371 |
| 29 | exp observational study/ | 147838 |
| 30 | exp clinical trial/ | 1942227 |
| 31 | clinical study/ | 100555 |
| 32 | exp Evaluation Studies/ | 268804 |
| 33 | exp Evaluation Studies as Topic/ | 1043035 |
| 34 | exp quantitative study/ | 27783 |
| 35 | exp validation studies/ | 147400 |
| 36 | exp experimental study/ | 21411 |
| 37 | exp quasi experimental study/ | 3765 |
| 38 | exp field study/ | 7416 |
| 39 | in vivo study/ | 256733 |
| 40 | exp panel study/ | 617 |
| 41 | exp Pilot Projects/ | 224511 |
| 42 | exp pilot study/ | 224511 |
| 43 | exp prevention study/ | 3186 |
| 44 | exp replication study/ | 1576 |
| 45 | exp theoretical study/ | 1590649 |
| 46 | exp Feasibility Studies/ | 135935 |
| 47 | exp trend study/ | 18944 |
| 48 | exp correlational study/ | 23181 |
| 49 | exp case-control studies/ | 1007383 |
| 50 | exp confidence interval/ | 160392 |
| 51 | exp regression analysis/ | 738355 |
| 52 | exp proportional hazards model/ | 137237 |
| 53 | exp multivariate analysis/ | 438118 |
| 54 | ((evidence adj based) or (outcome* adj (research or assessment*)) or (meta adj analys*) or (systematic* adj3 review*) or guideline* or (control* adj3 study) or (control* adj3 trial) or (randomized adj3 study) or (randomized adj3 trial) or (randomised adj3 study) or (randomised adj3 trial) or "pragmatic clinical trial" or (random* adj1 allocat*) or (doubl* adj blind*) or (doubl* adj mask*) or (singl* adj blind*) or (singl* adj mask*) or (tripl* adj blind*) or (tripl* adj mask*) or (trebl* adj blind*) or (trebl* adj mask*) or "latin square" or placebo* or nocebo* or random* or control* or multivariate or "comparative study" or "comparative survey" or "comparative analysis" or compar* or (intervention* adj2 study) or (intervention* adj2 trial) or "cross-sectional study" or "cross-sectional analysis" or "cross-sectional survey" or "cross-sectional design" or "prevalence study" or "prevalence analysis" or "prevalence survey" or "disease frequency study" or "disease frequency analysis" or "disease frequency survey" or crossover or "cross-over" or cohort* or "longitudinal study" or "longitudinal survey" or "longitudinal analysis" or "longitudinal evaluation" or longitudinal* or ((retrospective or "ex post facto") adj3 (study or survey or analysis or design)) or retrospectiv* or "prospective study" or "prospective survey" or "prospective analysis" or prospectiv* or (population adj3 (stud* or survey* or analys* or research)) or "concurrent study" or "concurrent survey" or "concurrent analysis" or "incidence study" or "incidence survey" or "incidence analysis" or (("follow-up" or followup) adj (stud* or survey or analysis)) or ((observation or observational) adj (study or survey or analysis)) or "case study" or "case series" or "clinical series" or "case studies" or "clinical study" or "clinical trial" or "evaluation study" or "evaluation survey" or "evaluation analysis" or "quantitative study" or "quantitative analys*" or "numerical study" or "validation study" or "validation survey" or "validation analysis" or "experimental study" or "experimental analysis" or "quasi experimental study" or "quasi experimental analysis" or "quasiexperimental study" or "quasiexperimental analysis" or "field study" or "field survey" or "field analysis" or "in vivo study" or "in vivo analysis" or "panel study" or "panel survey" or "panel analysis" or "pilot study" or "pilot survey" or "pilot analysis" or "pilot project" or ((prevention or preventive) adj3 (trial or study or analysis or survey)) or "replication study" or "replication analysis " or "replication trial" or "theoretical study" or "theoretical analysis " or "feasibility study" or "feasibility analysis" or "trend study" or "trend survey" or "trend analysis" or ((correlation* adj2 study) or (correlation* adj2 analys*)) or "case control study" or "case base study" or "case referrent study" or "case referent study" or "case referent study" or "case compeer study" or "case comparison study" or "matched case control" or "multicenter study" or "multi-center study" or study or trial or pilot or "odds ratio" or "confidence interval" or "regression analysis" or "least square" or "least squares" or (hazard* adj (model* or analys* or regression or ratio or ratios)) or "Cox model" or "Cox multivariate analyses" or "Cox multivariate analysis" or "Cox regression" or "Cox survival analyses" or "Cox survival analysis" or "Cox survival model" or "change analysis" or review).mp,pt. | 30778332 |
| 55 | or/7-54 | 31565509 |
| 56 | 6 and 55 | 1632 |
| 57 | from 6 keep 1539-2205 | 667 |
| 58 | limit 57 to (clinical trial, all or clinical trial, phase i or clinical trial, phase ii or clinical trial, phase iii or clinical trial, phase iv or clinical trial or controlled clinical trial or multicenter study or observational study or randomized controlled trial or pragmatic clinical trial or comparative study or controlled clinical trial or evaluation studies or guideline or practice guideline or meta analysis or multicenter study or observational study or randomized controlled trial or pragmatic clinical trial or systematic reviews or validation studies) [Limit not valid in Embase,CCTR,CDSR; records were retained] | 141 |
| 59 | 56 or 58 | 1632 |
| 60 | limit 59 to english language [Limit not valid in CDSR; records were retained] | 1541 |
| 61 | limit 60 to yr="1990 -Current" | 1505 |
| 62 | limit 61 to (conference abstract or editorial or erratum or letter or note or addresses or autobiography or bibliography or biography or blogs or comment or dictionary or directory or interactive tutorial or interview or lectures or legal cases or legislation or news or newspaper article or overall or patient education handout or periodical index or portraits or published erratum or video-audio media or webcasts) [Limit not valid in Embase,CCTR,CDSR,Ovid MEDLINE(R),Ovid MEDLINE(R) Daily Update,Ovid MEDLINE(R) In-Process,Ovid MEDLINE(R) Publisher; records were retained] | 397 |
| 63 | 61 not 62 | 1108 |
| 64 | remove duplicates from 63 | 767 |

Scopus

1. TITLE-ABS-KEY(((cooking or "food preparation" or culinary) W/3 (class* or instruction or education or intervention* or program* or course* or workshop*)) OR "teaching kitchen")
2. TITLE-ABS-KEY((evidence W/1 based) OR (outcome* W/1 (research OR assessment*)) OR (meta W/1 analys*) OR (systematic* W/3 review*) OR guideline* OR (control* W/3 study) OR (control* W/3 trial) OR (randomized W/3 study) OR (randomized W/3 trial) OR (randomised W/3 study) OR (randomised W/3 trial) OR "pragmatic clinical trial" OR (random* W/1 allocat*) OR (doubl* W/1 blind*) OR (doubl* W/1 mask*) OR (singl* W/1 blind*) OR (singl* W/1 mask*) OR (tripl* W/1 blind*) OR (tripl* W/1 mask*) OR (trebl* W/1 blind*) OR (trebl* W/1 mask*) OR "latin square" OR placebo* OR nocebo* OR random* OR control* OR multivariate OR "comparative study" OR "comparative survey" OR "comparative analysis" OR compar* OR (intervention* W/2 study) OR (intervention* W/2 trial) OR "cross-sectional study" OR "cross-sectional analysis" OR "cross-sectional survey" OR "cross-sectional design" OR "prevalence study" OR "prevalence analysis" OR "prevalence survey" OR "disease frequency study" OR "disease frequency analysis" OR "disease frequency survey" OR crossover OR "cross-over" OR cohort* OR "longitudinal study" OR "longitudinal survey" OR "longitudinal analysis" OR "longitudinal evaluation" OR longitudinal* OR ((retrospective OR "ex post facto") W/3 (study OR survey OR analysis OR design)) OR retrospectiv* OR "prospective study" OR "prospective survey" OR "prospective analysis" OR prospectiv* OR (population W/3 (stud* or survey* or analys* or research)) OR "concurrent study" OR "concurrent survey" OR "concurrent analysis" OR "incidence study" OR "incidence survey" OR "incidence analysis" OR (("follow-up" or followup) W/1 (stud* or survey or analysis)) OR ((observation or observational) W/1 (study or survey or analysis)) OR "case study" OR "case series" OR "clinical series" OR "case studies" OR "clinical study" OR "clinical trial" OR "evaluation study" OR "evaluation survey" OR "evaluation analysis" OR "quantitative study" OR "quantitative analys*" OR "numerical study" OR "validation study" OR "validation survey" OR "validation analysis" OR "experimental study" OR "experimental analysis" OR "quasi experimental study" OR "quasi experimental analysis" OR "quasiexperimental study" OR "quasiexperimental analysis" OR "field study" OR "field survey" OR "field analysis" OR "in vivo study" OR "in vivo analysis" OR "panel study" OR "panel survey" OR "panel analysis" OR "pilot study" OR "pilot survey" OR "pilot analysis" OR "pilot project" OR ((prevention or preventive) W/3 (trial or study or analysis or survey)) OR "replication study" OR "replication analysis " OR "replication trial" OR "theoretical study" OR "theoretical analysis " OR "feasibility study" OR "feasibility analysis" OR "trend study" OR "trend survey" OR "trend analysis" OR ((correlation* W/2 study) OR (correlation* W/2 analys*)) OR "case control study" OR "case base study" OR "case referrent study" OR "case referent study" OR "case referent study" OR "case compeer study" OR "case comparison study" OR "matched case control" OR "multicenter study" OR "multi-center study" OR study OR trial OR pilot OR "odds ratio" OR "confidence interval" OR "regression analysis" OR "least square" OR "least squares" OR (hazard* W/1 (model* OR analys* OR regression or ratio or ratios)) OR "Cox model" OR "Cox multivariate analyses" OR "Cox multivariate analysis" OR "Cox regression" OR "Cox survival analyses" OR "Cox survival analysis" OR "Cox survival model" OR "change analysis" OR review)
3. PUBYEAR AFT 1989 AND LANGUAGE(english)
4. 1 and 2 and 3
5. DOCTYPE(le) OR DOCTYPE(ed) OR DOCTYPE(bk) OR DOCTYPE(er) OR DOCTYPE(no) OR DOCTYPE(sh) OR DOCTYPE(ab)
6. 4 and not 5
7. PMID(0*) OR PMID(1*) OR PMID(2*) OR PMID(3*) OR PMID(4*) OR PMID(5*) OR PMID(6*) OR PMID(7*) OR PMID(8*) OR PMID(9*)
8. 6 and not 7

# *Supplemental Tables*

## *Table 1: Studies Characteristics*

| Author, Year, Country | Population (Age Group) | Study Objectives | Duration (weeks) | Intervention Description | Class instructor | Is cooking participatory? | Outcome Description | Sample Size | Mean Age (years) |
| --- | --- | --- | --- | --- | --- | --- | --- | --- | --- |
| Archuleta, 2012[[1](#_ENREF_1)] USA | Adults | Determining whether cooking classes improve nutrient intake patterns in people with T2DM. | 4 | Four weekly meetings for 3 hours each consisting of nutrition recommendations for people with type 2 diabetes and hands-on cooking. | Dietitian | Yes | Changes in dietary intake. | Intervention: 117 | Intervention: 63 |
| Barak-Nahum, 2016[[2](#_ENREF_2)] Israel | Adults | Effect of culinary intervention on cancer patients' quality of life through changes in eating behavior. | 10 | Two hour long meetings addressing a particular nutrition topic, and involving a “hands-on” cooking session, followed by group discussion. | Dietitian | Yes | Health related quality of life, subjective well-being (negative affect, positive affect), and eating behaviors (permission to eat, eating for physical reasons, reliance on hunger and healthy food choices) | Intervention: 96 Control: 88 | Intervention: 57.6 Control: 58.1 |
| Burrows, 2015[[3](#_ENREF_3)] Australia | Children | Evaluation of the impact of a cooking club on dietary behaviors in a population at risk of obesity at a low income school. | 10 | Five 90-minute cooking sessions after-school, once every 2 weeks. | Dietitian | Yes | Changes in social cognitive theory constructs and dietary behavior. | Intervention: 51 | Intervention: 9 |
| Caraher, 2012[[4](#_ENREF_4)] UK | Children | Assessing the impact of a cooking class on food preparation, consumption and cooking confidence. | 52 | Two sessions covering hand washing, healthy eating, experiencing food through the senses and a practical cooking session. | Chef | Unclear | Students' confidence in food preparation and vegetable consumption. | Intervention: 86 Control: 83 | NR |
| Chen, 2014[[5](#_ENREF_5)] USA | Children | Assessing the impact of a cooking intervention among ethnically diverse elementary-school children and their family members. | 13 | Three in-class cooking demonstrations, tasting activities and providing students with the materials needed to prepare the same recipe at home with help from their parents. | Educator | No | Change in familiarity of and preference for the featured vegetables, parents' reported fruit and vegetable consumption of the children, child involvement in food preparation, and frequency of eating a meal together. | Intervention: 604 Control: 378 | Intervention: 6 Control: 6.1 |
| Cunningham-Sabo, 2013[[6](#_ENREF_6)] USA | Children | Assessing the effect of Cooking with Kids program. | 10 | 1-hour introductory lesson, three 2-hour cooking classes, and three 1-hour fruit and vegetable tasting sessions. | Educator | Unclear | Changes in preference for fruits and vegetables, attitude toward food and cooking and cooking self-efficacy. | Intervention: 137 Control: 120 | NR |
| Cunningham-Sabo, 2014[[7](#_ENREF_7)] USA | Children | Comparing effects of a cooking and tasting curriculum with a tasting-only curriculum on fourth graders’ cooking self-efficacy, cooking attitudes, and fruit and vegetable preferences. | 26 | Five 2-hour long cooking and/or five 1-hour fruit and vegetable tasting lessons throughout the school year. | Educator | Yes | Changes in self-efficacy, attitude, and preference. | Intervention: 539 Control: 397 | NR |
| Curtis, 2012[[8](#_ENREF_8)] UK | Children and Adults | Evaluation of the effects of interventions aimed at increasing intake of low-fat starchy foods. | 6 | Four "cook and eat" sessions each lasting for 2 hours, which focused on meal planning, food preparation and cooking skills required to incorporate more low-fat starchy foods into meals and snacks. | Dietitian | Yes | Changes in dietary intake. | Intervention: 108 Adults & 99 Children Control: 97 Adults & 92 Children | Intervention: 8.8 Control: 9.1 |
| D'Adamo, 2016[[9](#_ENREF_9)] USA | Children | Determining if nutrition education intervention improves diet quality and healthy eating. | 6 | Six one-hour long cooking sessions. | Chef | Yes | Change of mean intake of dietary components and change in attitudes toward healthy eating. | Intervention: 55 Control: 55 | Intervention: 16.2 Control: 17.1 |
| Flynn, 2013[[10](#_ENREF_10)] USA | Adults | Assessing if cooking classes increase vegetable consumption, improve food security and not adversely change body weight. | 6 | Cooking demonstrations and 6 months of follow-up that involved one appointment per month. | NR | No | Changes in dietary intake, food purchases, food security (average dollars/ week spent on food), and anthropometrics. | Intervention: 63 | Intervention: 51.8 |
| Gatto, 2017[[11](#_ENREF_11)] USA | Children | Exploring the effects of gardening, nutrition and cooking intervention on dietary intake, obesity parameters and metabolic disease risk. | 12 | Twelve 45 minute interactive cooking / nutrition lessons and 45 minute gardening lessons. | Educator | Yes | Anthropometrics and metabolic outcomes and Nutrients intake. | Intervention: 172 Control: 147 | Intervention: 9.3 Control: 9.3 |
| Gibbs, 2013[[12](#_ENREF_12)] Australia | Children | Evaluation of the program impact on children’s willingness to try new foods, capacity to describe foods, and healthy eating. | 104 | Weekly 45 to 60 minutes in a garden class with a garden specialist and 90 minutes in a kitchen class. | Chef | Yes | Willingness to try foods, Food choices and food descriptions, food and beverage intake. | Intervention: 463 Control: 280 | NR |
| Harmon, 2015[[13](#_ENREF_13)] USA | Children and Adults | Assessing the impact of culinary skills sessions on child involvement in meal preparation and changes in attitudes and self-efficacy related to cooking. | 4 | Four 1-hour long culinary skills training sessions once per week. Each session focused on teaching culinary skills and incorporating whole grains, fruits, and vegetables into each recipe. | NR | Yes | Children’s attitudes and self-efficacy related to cooking. | Children: 65 Adults: 25 (Pre-post) | NR |
| Herbert, 2014[[14](#_ENREF_14)] Australia | Adults | Assessing whether a cooking skills program has an impact on healthy cooking and eating and whether there are additional social and health benefits. | 10 | Ten 90-minute long cooking classes. | Chef | Unclear | Change in questionnaire answers targeting food purchasing behavior and attitudes, cooking and healthy eating knowledge, cooking enjoyment and satisfaction, social eating, and anthropometrics. | Intervention: 694 Control: 237 | NR |
| Kerrison, 2017[[15](#_ENREF_15)] USA | Adults | Evaluating effectiveness of a culinary nutrition program on knowledge, attitudes and self-efficacy. | 6 | Five three-hour long cooking demonstration sessions and grocery store tours. | Chef | No | Availability and accessibility of fruits and vegetables, cooking attitudes, cooking behaviors, cooking self-efficacy. | Intervention: 52 Control: 54 | NR |
| Kitaoka, 2013[[16](#_ENREF_16)] Japan | Adults | Evaluation of the effect of a dietary educational program for hypertensive men. | 22 | Five monthly 4 hour long session that consisted of a lecture and cooking instructions. | Dietitian | Yes | Changes in dietary habits, anthropometrics, and labs. | Intervention: 38 Control: 26 | Intervention: 66.2 Control: 64.1 |
| Levy, 2004[[17](#_ENREF_17)] USA | Adults | Determining if cooking classes improve subjects knowledge, attitudes and behaviors toward cooking. | 4 | Four 2-hour long basic cooking skills classes and a 45-minute supermarket tour. | Chef | Yes | Changes in attitudes, behavior and knowledge about cooking. | Intervention: 33 Control: 32 | Intervention: 19.6 Control: 19.8 |
| Liquori, 1998[[18](#_ENREF_18)] USA | Children | Examining the feasibility and effectiveness of a nutrition education intervention. | 26 | Ten weekly cooking classes. | Educator | Yes | Dietary intake, preference for plant foods, knowledge, and self-efficacy. | NR | NR |
| McKellar, 2007[[19](#_ENREF_19)] UK | Adults | Evaluating effects of cooking classes on quality of life for patients with RA and their consumption of healthier foods. | 6 | Six weekly 2-hour cooking sessions backed up with written information. | Dietitian | Yes | Changes in dietary intake, disease activity scores, CV risk factors, labs and anthropometrics. | Intervention: 75 Control: 55 | Intervention: 55 Control: 53 |
| Monlezun, 2015[[20](#_ENREF_20)] USA | Adults | Assessing biometrics using a cooking and nutrition curriculum for patients with T2DM. | 6 | Two-hour cooking class consisting of 30 minutes of didactic lessons and 90 minutes of cooking time. | Chef | Yes | Metabolic and lab outcomes, anthropometric and psychometric outcomes. | Intervention: 18 Control: 9 | Intervention: 62 |
| Newman, 2005[[21](#_ENREF_21)] USA | Adults | Evaluating effects of cooking classes on dietary change. | 52 | An orientation meeting, telephone counseling, newsletters, and 12 monthly cooking classes. | Dietitian | Unclear | Achievement of intervention dietary goals. | Intervention: 739 | Intervention: 54 |
| Quinn, 2003[[22](#_ENREF_22)] USA | Children | Assessing the impact of cooking classes on dietary habits and attitudes. | 52 | 11 sessions of cookshop program which consisted of 3 components that include the classroom, parental and school lunch intervention. | NR | Yes | Change in dietary consumption. | Intervention: 81 Control: 68 | Intervention: 10.7 Control: 10.5 |
| Robson, 2016[[23](#_ENREF_23)] USA | Children and Adults | Investigating the impact of a parent-child dyad cooking intervention on reducing eating dinner away from home. | 10 | 10 weekly cooking sessions lasting 60-90 minutes. | Dietitian | Yes | Changes in energy intake and diet quality including fat, saturated fat, Na and cholesterol, attitude and confidence toward cooking, and anthropometric measurements. | Adults: 6 Children: 6 (Pre-post) | Adults: 34.7 Children: 5.7 |
| Roche, 2017[[24](#_ENREF_24)] Ecuador | Children | Assessing effects of cooking and nutrition education on infant and young children's nutrition. | 2 | 12 basic nutrition and healthy recipe preparation sessions. | Educator | Yes | Mean percentage of recommended dietary intake. | Intervention: 80 Control: 184 | Intervention: 1.2 Control: 0.9 |
| Sorensen, 2011[[25](#_ENREF_25)] Denmark | Adults | Evaluation of weight regain behavior modification consisting of either a gourmet cooking course or neurolinguistic programming therapy. | 22 | Ten sessions aimed at preparing low-fat meals in which fat was replaced by flavoring with herbs, spices and condiments. | Dietitian | Yes | Participants who lost >10% weight, 5-10%, <5% or gained weight. | Intervention: 25 Control: 23 | Intervention: 40 Control: 42 |
| Takada, 2016[[26](#_ENREF_26)] Japan | Adults | Investigating whether cooking classes, focusing on salt reduction, given to housewives would influence their own and family members' salt consumption. | 9 | Two 90 minutes cooking classes focusing on salt reduction. | Dietitian | Unclear | Changes in estimated daily salt intake, and blood pressure. | Intervention: 36 Control: 32 | Intervention: 63 Control: 64.8 |
| Warmin, 2012[[27](#_ENREF_27)] USA | Adults | Evaluating effects of a culinary nutrition program on self-efficacy and healthy eating behaviors among college students. | 15 | Five 2-hour long cooking sessions. | Chef | Yes | Availability and accessibility of fruits and vegetables, cooking attitudes, cooking behaviors, cooking self-efficacy. | Intervention: 37 Control: 24 | NR |
| Waswa, 2015[[28](#_ENREF_28)] Kenya | Children and Adults | Assessing the effect of an educational intervention on children’s dietary diversity and nutrition knowledge of caregivers. | 22 | Four 2.5 hours nutrition education sessions included group trainings and cooking demonstrations. | NR | Yes | Anthropometrics and nutritional status for children, and anthropometrics and mean nutrition knowledge score for adults. | Intervention: 110 Control: 97 | Intervention: 1.2 & 26.6 Control: 1.1 & 25.1 |
| Watt, 2015[[29](#_ENREF_29)] USA | Adults | Evaluation of a primary care-based nutrition intervention targeting low-income Hispanic pregnant women in their first trimester. | 87 | Six cooking classes offered during the prenatal period and one baby food cooking class offered at 4–6 months postpartum. Each class lasted 1.5 hours. | Educator | Unclear | Mean change in maternal diet, exercise days/week, alcohol use, positive depression screen, stress, social support, maternal weight gain during pregnancy, breastfeeding status at 6months and child developmental outcomes. | Intervention: 32 Control: 29 | Intervention: 28.69 Control: 26.7 |
| Zahr, 2017[[30](#_ENREF_30)] Canada | Children | Evaluation of the influence of cooking class on students' food preferences, cooking skills and confidence. | Unclear | 4–5 sessions (2.5 hours each) teaching basic food and kitchen safety rules, knife skills, and how to make meals and snacks from scratch. | Chef | Yes | Change in food preference scores, changes observed at home by parents and willingness to try new food. | Intervention: 68 Control: 32 | NR |

## *Table 2: Outcomes reported in individual studies*

| First Author, Year, Country | Population (Age Group) | Sample Size | Follow-up Duration (weeks) | Outcome Description |
| --- | --- | --- | --- | --- |
| Archuleta, 2012[[1](#_ENREF_1)] USA | Adults | Intervention: 117 | 4 | There was a significant decrease in intakes of energy, fat grams, percentage of calories from fat, saturated fat grams, cholesterol, sodium, and carbohydrates after the culinary intervention. |
| Barak-Nahum, 2016[[2](#_ENREF_2)] Israel | Adults | Intervention: 96 Control: 88 | 10 | Participants in the culinary intervention group a significant higher health-related quality of life, and a significantly lower negative affect and higher positive affect. There was a significant main effect of study group on food choices and eating behaviors, such that participants in the culinary intervention group ate healthier foods than those in the wait-list group and had better eating behaviors in unconditional permission to allow oneself to eat, eating for physical reasons, and reliance on hunger cues. |
| Burrows, 2015[[3](#_ENREF_3)] Australia | Children | Intervention: 51 | 13 | Consumption of one or more fruit servings per day significantly increased and there was a trend for increasing the weekly variety of fruit and vegetables. The SCT constructs assessed within the current study improved significantly after the culinary intervention. |
| Caraher, 2012[[4](#_ENREF_4)] UK | Children | Intervention: 86 Control: 83 | 4 | Average reported cooking confidence score significantly increased in the culinary intervention group. In the control group this change was not statistically significant. Children’s average reported vegetable consumption significantly increased, with no significant changes in the control group. |
| Chen, 2014[[5](#_ENREF_5)] USA | Children | Intervention: 604 Control: 378 | 13 | Intervention students significantly increased their involvement in food preparation at home. |
| Cunningham-Sabo, 2013[[6](#_ENREF_6)] USA | Children | Intervention: 137 Control: 120 | 10 | Increases in vegetable preference, cooking attitude and self-efficacy were all significantly greater in the culinary intervention group. |
| Cunningham-Sabo, 2014[[7](#_ENREF_7)] USA | Children | Intervention: 539 Control: 397 | 52 | Fruit and Vegetable Preferences significantly improved in males. Students without cooking experience (61% male) had a significantly improved cooking attitude response. |
| Curtis, 2012[[8](#_ENREF_8)] UK | Children and Adults | Intervention: 108 Adults & 99 Children Control: 97 Adults & 92 Children | 78 | Individuals in the control group consumed significantly more starch than individuals in the culinary intervention group. Whereas, individuals in the culinary intervention group had significantly higher intakes of total carbohydrate than those in the control group. |
| D'Adamo, 2016[[9](#_ENREF_9)] USA | Children | Intervention: 55 Control: 55 | 10 | There were significant improvements in the culinary intervention group compared with control in whole grains (31.2 g/wk) and protein foods (13.2 ounces per week) intake, and attitudes toward eating vegetables, whole grains, lean protein, and low-fat dairy. |
| Flynn, 2013[[10](#_ENREF_10)] USA | Adults | Intervention: 63 | 26 | Total variety of vegetables and fruit intake significantly increased. Grocery receipts showed a significant decrease in purchases of meat, carbonated beverages, desserts, snacks and total groceries. Food Insecurity Score significantly decreased from baseline to follow-up, as did BMI. |
| Gatto, 2017[[11](#_ENREF_11)] USA | Children | Intervention: 172 Control: 147 | 2 | The culinary intervention group compared with controls had significantly greater reductions in BMI z-score and waist circumference. The culinary intervention group compared with controls also had a significant improvement in dietary fiber intake. All participants decreased vegetable intake, but decreases were significantly less in the culinary intervention group than controls. Change in fruit intake did not differ between the two groups. Fewer culinary intervention participants had the metabolic syndrome after the intervention than before, while controls with metabolic syndrome increased, but the change was not significant. |
| Gibbs, 2013[[12](#_ENREF_12)] Australia | Children | Intervention: 463 Control: 280 | 17 | Child and parent qualitative and quantitative measures (odds ratio 2.0; confidence interval, 1.06–3.58) showed significant increases in children’s reported willingness to try new foods. |
| Harmon, 2015[[13](#_ENREF_13)] USA | Children and Adults | Children: 65 Adults: 25 (Pre-post) | 4 | A statistically significant increase was seen in participant’s perceived cooking skills and abilities from pre to post-intervention. |
| Herbert, 2014[[14](#_ENREF_14)] Australia | Adults | Intervention: 694 Control: 237 | 26 | BMI did not significantly change after program attendance. There was a significant reduction of take away/fast food weekly purchasing, and significant increases in eating meals at the dinner table, cooking satisfaction, and the ability to prepare a meal in 30 minutes and from basics that were low in cost. |
| Kerrison, 2017[[15](#_ENREF_15)] USA | Adults | Intervention: 52 Control: 54 | post-intervention | Cooking Self-efficacy index/scale identified a significant difference between the culinary intervention and control group. Cooking attitude mean change of the culinary intervention vs comparison is not statistically significant. |
| Kitaoka, 2013[[16](#_ENREF_16)] Japan | Adults | Intervention: 38 Control: 26 | 22 | In the culinary intervention group, a significant decrease in the urinary sodium-to-potassium excretion ratio was observed, compared with the control group. The systolic and diastolic blood pressure significantly decreased in the culinary intervention group and no changes were observed in the control group. |
| Levy, 2004[[17](#_ENREF_17)] USA | Adults | Intervention: 33 Control: 32 | 13 | Analysis revealed no gender differences. The culinary intervention group experienced more statistically significant gains in attitudes and displayed positive, but not statistically significant, shifts in knowledge and some behaviors. |
| Liquori, 1998[[18](#_ENREF_18)] USA | Children | NR | post-intervention | Culinary intervention did not have an impact on cooking attitude, but had a positive impact on self-efficacy in cooking in the older children. |
| McKellar, 2007[[19](#_ENREF_19)] UK | Adults | Intervention: 75 Control: 55 | 26 | Significant benefit was shown in the culinary intervention group compared with controls for patient global assessment at 6 months, pain score at 3 and 6 months, early morning stiffness at 6 months and Health Assessment Questionnaire score at 3 months. There were significant increases in weekly total fruit, vegetable and legume consumption and improvement in the ratio of monounsaturated: saturated fat intake and systolic BP in the culinary intervention group only. |
| Monlezun, 2015[[20](#_ENREF_20)] USA | Adults | Intervention: 18 Control: 9 | 26 | Compared to the control group, the culinary intervention group had superior HbA1c reduction that was not statistically significant. There were significantly greater reductions in the culinary intervention vs. control group for DBP and total cholesterol. There was a greater proportion increase though not significant of culinary intervention subjects compared to controls who mostly believed they could eat correct portions, and who used nutrition panels to make food choices. Culinary intervention patients’ psychometrics improved but without statistical significance compared to the control group in their attitudes and competencies in healthy food shopping and eating. |
| Newman, 2005[[21](#_ENREF_21)] USA | Adults | Intervention: 739 | 52 | Total daily vegetable, vegetable juice, fruit, and fiber intake increased significantly, while fat decreased significantly after the culinary intervention. |
| Quinn, 2003[[22](#_ENREF_22)] USA | Children | Intervention: 81 Control: 68 | 52 | Children did not change their dietary habits, but they showed a difference in food exposure and willingness to try new foods. |
| Robson, 2016[[23](#_ENREF_23)] USA | Children and Adults | Adults: 6 Children: 6 (Pre-post) | 4 | The proportion of dinners consumed by parent-child dyads away from home significantly decreased after the culinary intervention. Dyad cholesterol intake at dinner also significantly decreased over time; however, changes in energy intake, total fat, saturated fat, and sodium at dinner were not significant. A large effect size was found for changes in parent ratings of enjoyment of cooking between baseline and post-treatment. A decrease over time was found, with small and medium effect sizes for child z-BMI (d= -0.22) and BMI percentile (d= -0.43), respectively. A medium effect size was found for change in parent BMI from baseline to post-treatment (d= -0.49). |
| Roche, 2017[[24](#_ENREF_24)] Ecuador | Children | Intervention: 80 Control: 184 | 26 | Mothers in the culinary intervention group were significantly more likely to feed their children the promoted foods. Children in the culinary intervention consumed a significantly higher percentage of recommended intakes for iron, zinc, vitamin A, protein, and energy at follow-up and had significant improvements in weight-for-age z-score. Likelihood of underweight was reduced for children in the culinary intervention group (OR=0.36; 95% confidence interval, 0.13–0.96). |
| Sorensen, 2011[[25](#_ENREF_25)] Denmark | Adults | Intervention: 25 Control: 23 | 156 | There was no difference in weight maintenance after two and three years of follow-up between the culinary intervention group and NLP therapy control group. |
| Takada, 2016[[26](#_ENREF_26)] Japan | Adults | Intervention: 36 Control: 32 | 9 | The mean daily salt intake in the culinary intervention group decreased significantly compared to controls (95% CI: -2.29,-0.09; P=0.034). A similar tendency was observed in the subgroups of housewives and family members. In both groups, systolic and diastolic blood pressure increased from baseline to the end of the trial, while the increases were less in the culinary intervention group, the difference was not statistically significant. |
| Warmin, 2012[[27](#_ENREF_27)] USA | Adults | Intervention: 37 Control: 24 | post-intervention | Culinary intervention group scored significantly higher from pre- to posttest scores for the self-efficacy scale. For the cooking attitude scale, there were no significant differences in any of the groups. |
| Waswa, 2015[[28](#_ENREF_28)] Kenya | Children and Adults | Intervention: 110 Control: 97 | 52 | The children’s dietary diversity scores (CDDS) improved significantly in the culinary intervention group at endline. The intervention also had a significant effect on the caregivers’ nutrition knowledge scores. |
| Watt, 2015[[29](#_ENREF_29)] USA | Adults | Intervention: 32 Control: 29 | 52 | Women in the culinary intervention group were more likely than women in the comparison group to have significant improvements in diet, exercise, and depression. In addition, participants were more likely to breastfeed (p=.07) and their infants were more likely to pass the ages and stages developmental screen (p=.06) than women in the comparison group. |
| Zahr, 2017[[30](#_ENREF_30)] Canada | Children | Intervention: 68 Control: 32 | 3 | Students in the culinary intervention group reported a significant increase in the familiarity and preference for the foods introduced through the program. They also reported a significant increase in cooking skills, making a balanced meal on their own and in confidence making the recipes introduced in the program. |

## *Table 3: Risk of bias assessment, randomized trials*

| Author, Year | Study Design | Selection: Random Sequence Generation: A) Low Risk B) High Risk C) Unclear | Selection: Allocation Concealment: A) Low Risk B) High Risk C) Unclear | Outcomes: Blinding of Participants or personnel: A) Low Risk B) High Risk C) Unclear | Outcomes: Blinding of Outcome Assessment: A) Low Risk B) High Risk C) Unclear | Outcomes: Incomplete Outcome Data: A) Low Risk B) High Risk C) Unclear | Outcomes: Reporting Bias/ Selective Reporting: A) Low Risk B) High Risk C) Unclear | Other Sources of Bias: A) Low Risk B) High Risk C) Unclear | Risk of Bias |
| --- | --- | --- | --- | --- | --- | --- | --- | --- | --- |
| Curtis, 2012[[8](#_ENREF_8)] | Cluster RCT | C | C | C | C | B | A | C | High |
| Gatto, 2017[[11](#_ENREF_11)] | Cluster RCT | C | B | B | B | A | A | B | High |
| Monlezun, 2015[[20](#_ENREF_20)] | RCT | C | C | B | C | A | A | C | High |
| Sorensen, 2011[[25](#_ENREF_25)] | RCT | C | C | C | C | B | A | C | High |
| Takada, 2016[[26](#_ENREF_26)] | Cluster RCT | C | C | B | A | B | A | C | High |
| Waswa, 2015[[28](#_ENREF_28)] | Cluster RCT | C | C | B | C | A | A | C | High |

## *Table 4: Risk of bias assessment, nonrandomized studies*

| Author, Year | Study Design | Selection: Representativeness of the exposed cohort A) Truly representative B) Somewhat representative C) No description of the derivation of the cohort | Selection* of the non-exposed cohort: A) Drawn from the same community as the exposed cohort. B) Drawn from general population (matched for important criteria). C) No description of the derivation of the non-exposed cohort | Comparability of cohorts on the basis of the design or analysis (matching/ confounder adjustment): A) Study controls for age and sex. B) Study controls for other important factor. C) No description | Outcome: Assessment of outcome: A) Independent blind assessment. B) Record linkage. C) Standard procedure well described. D) Self report E) No description | Outcome: Was follow up long enough for outcomes to occur: A) Yes: >12w B) No: <12w | Outcome: Adequacy of follow up: A) Complete follow up- all subjects accounted for B) Subjects lost to follow up unlikely to introduce bias -small number lost- >80% follow up, or description of those lost. C) Follow up rate <80 % and no description of those lost. D) No statement | Risk of Bias |
| --- | --- | --- | --- | --- | --- | --- | --- | --- |
| Archuleta, 2012[[1](#_ENREF_1)] | Pre-post Study | B | N/A | C | D | B | B | High |
| Barak-Nahum, 2016[[2](#_ENREF_2)] | Nonrandomized Controlled Trial | B | A | C | D | B | B | High |
| Burrows, 2015[[3](#_ENREF_3)] | Pre-post Study | B | N/A | C | D | A | B | Moderate |
| Caraher, 2012[[4](#_ENREF_4)] | Nonrandomized Controlled Trial | A | A | C | D | B | D | Moderate |
| Chen, 2014[[5](#_ENREF_5)] | Nonrandomized Controlled Trial | A | A | C | D | A | D | Moderate |
| Cunningham-Sabo, 2013[[6](#_ENREF_6)] | Nonrandomized Controlled Trial | A | A | A | D | B | B | Moderate |
| Cunningham-Sabo, 2014[[7](#_ENREF_7)] | Nonrandomized Controlled Trial | A | A | A | D | A | D | Moderate |
| D'Adamo, 2016[[9](#_ENREF_9)] | Nonrandomized Controlled Trial | B | A | C | C | B | D | High |
| Flynn, 2013[[10](#_ENREF_10)] | Pre-post Study | B | N/A | C | C | A | A | Moderate |
| Gibbs, 2013[[12](#_ENREF_12)] | Nonrandomized Controlled Trial | A | A | C | D | A | C | Moderate |
| Harmon, 2015[[13](#_ENREF_13)] | Pre-post Study | A | N/A | C | D | B | A | Moderate |
| Herbert, 2014[[14](#_ENREF_14)] | Nonrandomized Controlled Trial | B | A | C | D | A | C | Moderate |
| Kerrison, 2017[[15](#_ENREF_15)] | Nonrandomized Controlled Trial | A | A | C | D | B | D | Moderate |
| Kitaoka, 2013[[16](#_ENREF_16)] | Nonrandomized Controlled Trial | B | A | C | C | A | B | High |
| Levy, 2004[[17](#_ENREF_17)] | Nonrandomized Controlled Trial | A | A | C | D | A | B | Moderate |
| Liquori, 1998[[18](#_ENREF_18)] | Nonrandomized Controlled Trial | A | A | C | D | B | D | Moderate |
| McKellar, 2007[[19](#_ENREF_19)] | Nonrandomized Controlled Trial | A | A | C | C | A | D | Moderate |
| Newman, 2005[[21](#_ENREF_21)] | Pre-post Study | B | N/A | C | D | A | D | High |
| Quinn, 2003[[22](#_ENREF_22)] | Pre-post Study | A | N/A | C | D | A | D | Moderate |
| Robson, 2016[[23](#_ENREF_23)] | Pre-post Study | A | N/A | C | D | B | A | Moderate |
| Roche, 2017[[24](#_ENREF_24)] | Nonrandomized Controlled Trial | B | A | C | C | A | D | Moderate |
| Warmin, 2012[[27](#_ENREF_27)] | Nonrandomized Controlled Trial | A | A | C | D | B | D | Moderate |
| Watt, 2015[[29](#_ENREF_29)] | Nonrandomized Controlled Trial | A | A | C | C | A | D | Moderate |
| Zahr, 2017[[30](#_ENREF_30)] | Nonrandomized Controlled Trial | C | A | C | D | B | B | High |

* Ascertainment of exposure (a component of the selection domain) was removed from the table because it was fulfilled in all of the studies

## *Table 5: Possible effect modifiers of the effect of interventions*

|  | OR | 95% CI | P value |
| --- | --- | --- | --- |
| **Self-efficacy** |  |  |  |
| Sample size | 1.04 | 0.95 - 1.14 | 0.35 |
| **Healthy intake** |  |  |  |
| Sample size | 1.03 | 0.99 - 1.08 | 0.12 |
| Class provider | 1.00 | 0.05 - 22.18 | 1.00 |
| Population (Children vs. Adults) | 1.50 | 0.07 - 31.57 | 0.79 |
| Number of sessions | 0.72 | 0.40 - 1.30 | 0.28 |
| Intervention duration | 0.99 | 0.92 - 1.05 | 0.66 |
| **Fruit and vegetable Intake** |  |  |  |
| Sample size | 1.00 | 1.00 - 1.01 | 0.59 |
| Population (Children vs. Adults) | 0.33 | 0.02 - 4.74 | 0.42 |
| Number of sessions | 0.68 | 0.41 - 1.13 | 0.13 |
| Intervention duration | 1.02 | 0.97 - 1.08 | 0.40 |

## *Table 6. Studies with intervention components in addition to cooking classes*

| **Study** | **Intervention Components** | **Outcomes significantly improved** |
| --- | --- | --- |
| D'Adamo, 2016[33] USA | Cooking, education and group activities | Dietary intake, AT |
| Gatto, 2015[34] USA | Cooking, education and gardening | BMI, dietary intake |
| Archuleta, 2012[14] USA | Cooking and education | Dietary intake |
| Barak-Nahum, 2016[15] Israel | Cooking, education and group activities | QoL, dietary intake |
| Burrows, 2015[28] Australia | Cooking and group activities | Dietary intake, SE |
| Caraher, 2012[29] UK | Cooking and education | SE, dietary intake |
| Cunningham-Sabo, 2014[32] USA | Cooking and education | AT |
| Curtis, 2012[40] UK | Cooking, education and goal setting | Dietary intake |
| Gibbs, 2013[35] Australia | Cooking and gardening | AT |
| Harmon, 2015[41] USA | Cooking and education | SE |
| Kerrison, 2017[18] USA | Cooking demonstration and grocery store tour | SE |
| Kitaoka, 2013[19] Japan | Cooking and education | BP |
| Levy, 2004[20] USA | Cooking and grocery store tour | AT |
| McKellar, 2007[21] UK | Cooking and education | QoL, dietary intake, BP |
| Monlezun, 2015[22] USA | Cooking and education | SE, AT |
| Newman, 2005[23] USA | Cooking and education | Dietary intake |
| Robson, 2016[42] USA | Cooking, education and group activities | Dietary intake, AT |
| Roche, 2017[38] Ecuador | Cooking and education | BMI, dietary intake |
| Waswa, 2015[43] Kenya | Cooking and education | Dietary intake |
| Watt, 2015[27] USA | Cooking, education and group activities | AT |
| Zahr, 2017[39] Canada | Cooking and education | SE, AT |

AT: attitudes, SE: self-efficacy, QoL: quality of life, BP: blood pressure, BMI: body mass index

# *References*

1. Archuleta M, Vanleeuwen D, Halderson K, Jackson K, Bock MA, Eastman W, Powell J, Titone M, Marr C, Wells L: **Cooking schools improve nutrient intake patterns of people with type 2 diabetes**. *J Nutr Educ Behav* 2012, **44**(4):319-325.

2. Barak-Nahum A, Haim LB, Ginzburg K: **When life gives you lemons: The effectiveness of culinary group intervention among cancer patients**. *Soc Sci Med* 2016, **166**:1-8.

3. Burrows TL, Lucas H, Morgan PJ, Bray J, Collins CE: **Impact Evaluation of an After-school Cooking Skills Program in a Disadvantaged Community: Back to Basics**. *Can J Diet Pract Res* 2015, **76**(3):126-132.

4. Caraher M, Seeley A, Wu M, Lloyd S: **When chefs adopt a school? An evaluation of a cooking intervention in English primary schools**. *Appetite* 2013, **62**:50-59.

5. Chen Q, Goto K, Wolff C, Bianco-Simeral S, Gruneisen K, Gray K: **Cooking up diversity. Impact of a multicomponent, multicultural, experiential intervention on food and cooking behaviors among elementary-school students from low-income ethnically diverse families**. *Appetite* 2014, **80**:114-122.

6. Cunningham-Sabo L, Lohse B: **Cooking with Kids positively affects fourth graders' vegetable preferences and attitudes and self-efficacy for food and cooking**. *Child* 2013, **9**(6):549-556.

7. Cunningham-Sabo L, Lohse B: **Impact of a school-based cooking curriculum for fourth-grade students on attitudes and behaviors is influenced by gender and prior cooking experience**. *J Nutr Educ Behav* 2014, **46**(2):110-120.

8. Curtis PJ, Adamson AJ, Mathers JC: **Effects on nutrient intake of a family-based intervention to promote increased consumption of low-fat starchy foods through education, cooking skills and personalised goal setting: the Family Food and Health Project**. *Br J Nutr* 2012, **107**(12):1833-1844.

9. D'Adamo CR, McArdle PF, Balick L, Peisach E, Ferguson T, Diehl A, Bustad K, Bowden B, Pierce BA, Berman BM: **Spice MyPlate**. *Am J Health Promot* 2016, **30**(5):346-356.

10. Flynn MM, Reinert S, Schiff AR: **A Six-Week Cooking Program of Plant-Based Recipes Improves Food Security, Body Weight, and Food Purchases for Food Pantry Clients**. *Journal of Hunger and Environmental Nutrition* 2013, **8**(1):73-84.

11. Gatto NM, Martinez LC, Spruijt-Metz D, Davis JN: **LA sprouts randomized controlled nutrition, cooking and gardening programme reduces obesity and metabolic risk in Hispanic/Latino youth**. *Pediatr Obes* 2017, **12**(1):28-37.

12. Gibbs L, Staiger PK, Johnson B, Block K, Macfarlane S, Gold L, Kulas J, Townsend M, Long C, Ukoumunne O: **Expanding children's food experiences: the impact of a school-based kitchen garden program**. *J Nutr Educ Behav* 2013, **45**(2):137-146.

13. Harmon BE, Smith N, Pirkey P, Beets MW, Blake CE: **The Impact of Culinary Skills Training on the Dietary Attitudes and Behaviors of Children and Parents**. *Am J Health Educ* 2015, **46**(5):283-292.

14. Herbert J, Flego A, Gibbs L, Waters E, Swinburn B, Reynolds J, Moodie M: **Wider impacts of a 10-week community cooking skills program--Jamie's Ministry of Food, Australia**. *BMC Public Health* 2014, **14**:1161.

15. Kerrison DA, Condrasky MD, Sharp JL: **Culinary nutrition education for undergraduate nutrition dietetics students**. *Br Food J* 2017, **119**(5):1045-1051.

16. Kitaoka K, Nagaoka J, Matsuoka T, Shigemura C, Harada K, Aoi W, Wada S, Asano H, Sakane N, Higashi A: **Dietary intervention with cooking instructions and self-monitoring of the diet in free-living hypertensive men**. *Clin Exp Hypertens* 2013, **35**(2):120-127.

17. Levy J, Auld G: **Cooking Classes Outperform Cooking Demonstrations for College Sophomores**. *Journal of Nutrition Education and Behavior* 2004, **36**(4):197-203.

18. Liquori T, Koch PD, Ruth Contento I, Castle J: **The Cookshop Program: Outcome Evaluation of a Nutrition Education Program Linking Lunchroom Food Experiences with Classroom Cooking Experiences**. *Journal of Nutrition Education* 1998, **30**(5):302-313.

19. McKellar G, Morrison E, McEntegart A, Hampson R, Tierney A, Mackle G, Scoular J, Scott JA, Capell HA: **A pilot study of a Mediterranean-type diet intervention in female patients with rheumatoid arthritis living in areas of social deprivation in Glasgow**. *Ann Rheum Dis* 2007, **66**(9):1239-1243.

20. Monlezun DJ, Kasprowicz E, Tosh KW, Nix J, Urday P, Tice D, Sarris L, Harlan TS: **Medical school-based teaching kitchen improves HbA1c, blood pressure, and cholesterol for patients with type 2 diabetes: Results from a novel randomized controlled trial**. *Diabetes Res Clin Pract* 2015, **109**(2):420-426.

21. Newman VA, Thomson CA, Rock CL, Flatt SW, Kealey S, Bardwell WA, Caan BJ, Pierce JP, Women's Healthy E, Living Study G: **Achieving substantial changes in eating behavior among women previously treated for breast cancer--an overview of the intervention**. *J Am Diet Assoc* 2005, **105**(3):382-391; quiz 488.

22. Quinn LJ, Horacek TM, Castle J: **The Impact of CookshopTM on the Dietary Habits and Attitudes of Fifth Graders**. *Topics in Clinical Nutrition* 2003, **18**(1):42-48.

23. Robson SM, Stough CO, Stark LJ: **The impact of a pilot cooking intervention for parent-child dyads on the consumption of foods prepared away from home**. *Appetite* 2016, **99**:177-184.

24. Roche ML, Marquis GS, Gyorkos TW, Blouin B, Sarsoza J, Kuhnlein HV: **A Community-Based Positive Deviance/Hearth Infant and Young Child Nutrition Intervention in Ecuador Improved Diet and Reduced Underweight**. *J Nutr Educ Behav* 2017, **49**(3):196-203 e191.

25. Sorensen LB, Greve T, Kreutzer M, Pedersen U, Nielsen CM, Toubro S, Astrup A: **Weight maintenance through behaviour modification with a cooking course or neurolinguistic programming**. *Can J Diet Pract Res* 2011, **72**(4):181-185.

26. Takada T, Imamoto M, Fukuma S, Yamamoto Y, Sasaki S, Uchida M, Miura Y, Shimizu S, Nihata K, Fukuhara S: **Effect of cooking classes for housewives on salt reduction in family members: a cluster randomized controlled trial**. *Public Health* 2016, **140**:144-150.

27. Warmin A, Sharp J, Condrasky MD: **Cooking with a chef: A culinary nutrition program for college aged students**. *Topics in Clinical Nutrition* 2012, **27**(2):164-173.

28. Waswa LM, Jordan I, Herrmann J, Krawinkel MB, Keding GB: **Community-based educational intervention improved the diversity of complementary diets in western Kenya: results from a randomized controlled trial**. *Public Health Nutr* 2015, **18**(18):3406-3419.

29. Watt TT, Appel L, Lopez V, Flores B, Lawhon B: **A Primary Care-Based Early Childhood Nutrition Intervention: Evaluation of a Pilot Program Serving Low-Income Hispanic Women**. *J Racial Ethn Health Disparities* 2015, **2**(4):537-547.

30. Zahr R, Sibeko L: **Influence of a School-Based Cooking Course on Students' Food Preferences, Cooking Skills, and Confidence**. *Can J Diet Pract Res* 2017, **78**(1):37-41.
